# Influence of Initial Temperature and Convective Heat Loss on the Self-Propagating Reaction in Al/Ni Multilayer Foils

**DOI:** 10.3390/ma14247815

**Published:** 2021-12-17

**Authors:** Mostafa Baloochi, Deepshikha Shekhawat, Sascha Sebastian Riegler, Sebastian Matthes, Marcus Glaser, Peter Schaaf, Jean Pierre Bergmann, Isabella Gallino, Jörg Pezoldt

**Affiliations:** 1 FG Nanotechnologie, Institut für Mikro-und Nanoelektronik, Institut für Mikro- und Nanotechnologien MacroNano®, Institut für Werkstofftechnik, TU Ilmenau, Postfach 100565, 98684 Ilmenau, Germany; deepshikha.shekhawat@tu-ilmenau.de; 2 Lehrstuhl für Metallische Werkstoffe, Universität des Saarlandes, Campus C6.3, 66123 Saarbrücken, Germany; sascha.riegler@uni-saarland.de (S.S.R.); i.gallino@mx.uni-saarland.de (I.G.); 3 FG Werkstoffe der Elektrotechnik, Institut für Werkstofftechnik, Institut für Mikro- und Nanotechnologien MacroNano®, TU Ilmenau, Gustav-Kirchhoff-Strasse 5, 98693 Ilmenau, Germany; sebastian.matthes@tu-ilmenau.de (S.M.); peter.schaaf@tu-ilmenau.de (P.S.); 4 FG Fertigungstechnik, Institut für Mikro- und Nanotechnologien MacroNano®, TU Ilmenau, Postfach 100565, 98684 Ilmenau, Germany; marcus.glaser@tu-ilmenau.de (M.G.); jeanpierre.bergmann@tu-ilmenau.de (J.P.B.)

**Keywords:** reactive materials, superlattice, nickel, aluminum, propagation velocity, self-sustained reaction, self-propagating reaction, transformation imprinted materials

## Abstract

A two-dimensional numerical model for self-propagating reactions in Al/Ni multilayer foils was developed. It was used to study thermal properties, convective heat loss, and the effect of initial temperature on the self-propagating reaction in Al/Ni multilayer foils. For model adjustments by experimental results, these Al/Ni multilayer foils were fabricated by the magnetron sputtering technique with a 1:1 atomic ratio. Heat of reaction of the fabricated foils was determined employing Differential Scanning Calorimetry (DSC). Self-propagating reaction was initiated by an electrical spark on the surface of the foils. The movement of the reaction front was recorded with a high-speed camera. Activation energy is fitted with these velocity data from the high-speed camera to adjust the numerical model. Calculated reaction front temperature of the self-propagating reaction was compared with the temperature obtained by time-resolved pyrometer measurements. X-ray diffraction results confirmed that all reactants reacted and formed a B2 NiAl phase. Finally, it is predicted that (1) increasing thermal conductivity of the final product increases the reaction front velocity; (2) effect of heat convection losses on reaction characteristics is insignificant, e.g., the foils can maintain their characteristics in water; and (3) with increasing initial temperature of the foils, the reaction front velocity and the reaction temperature increased.

## 1. Introduction

The Nickel and Aluminum binary system in different forms such as particles, powder, flakes, nanorods, and foils, were under intensive investigation since the end of the 20th century [1,2,3,4,5,6,7,8,9,10,11,12,13]. Fast and steady reaction and releasing a huge amount of energy on a very short time scale are the main beneficial aspects, applied in different fields of science and engineering. For example, applications are found in aerospace [14], thermal batteries [15], and potential application in drug delivery systems [16], where thermally actuated valving mechanism could be obtained by the development of reactive nanorods and porous thin films, and also at a smaller scale, such as joining electrical components in the semiconductor industry [17,18], welding [19], self-healing [20], etc. When the periodic bilayer thickness is less than 200 nm, the diffusion length decreases, and atomic diffusion will play a dominating role in the direction normal to the multilayer surface and thermal diffusion along the layers will be the governing process. Consequently, the chemical mixing rate increases, and in presence of external stimulation, the reaction occurs in the in-plane direction of the binary film in high-temperature self-propagating mode. In this case, theoretically, the temperature could increase up to 1912 K, which is the melting temperature of the NiAl B2 phase. The measured propagation velocity of the reaction front as reported in the literature is up to 15 m s^−1^, depending on the bilayer thickness and the intermixing at the interfaces between the layers [21,22].

Complexities and limitations of measurement devices and techniques drew attention towards analytical and numerical modeling of such reactions in multilayer foils [21,23,24,25,26,27,28]. In [21,29], the reaction front velocity was investigated in dependence on bilayer and intermixing at the interface between the layers and it was concluded that the average combustion velocity increases, when the periodic thickness decreases, but below a critical thickness, where the intermixing zone becomes comparable to the bilayer thickness, this trend is inverted. Former models showed that this prior intermixing in the multilayers decreased the reaction front velocity by reducing the rate of atomic diffusion [23]. Different techniques have been implemented to measure the heat of reaction and the activation energy of the self-propagating reaction [30,31,32,33]. In the current study, the activation energy was fitted to the measured reaction front velocity from high-speed camera measurement. The reaction energy was measured by differential scanning calorimetry (DSC). In [34], the effect of activation energy and initial temperature on the reaction front velocity and the reaction temperature in the Ti-C system was studied. In the simulations performed here, this effect is considered for the Al/Ni systems as well. In [27], a reduced model was introduced to investigate the reaction front velocity in direction normal to multilayers of uniform and non-uniform free-standing composite foils. In the current study, the effect of ambient conditions and the initial temperature of the foils on reaction characteristics were investigated. For this, a diffusion-limited finite element model was built with the help of COMSOL MULTIPHYSICS 5.6. To our knowledge, the influence of the initial temperature of nickel and aluminum multilayer foils on the characteristics of the reaction had not been investigated so far in the literature.

Due to different fabrication setups and environments, different microstructures are deposited, the main factors which are affecting the heat of reaction and consequently, the ignition properties of multilayer foils, are the bilayer thickness and premixing at the interface of the individual nickel and aluminum layers. The sputtering-induced alloying caused the formation of a few nanometer-thick NiAl intermetallic alloy at the interface of Ni and Al, which can act as a diffusion barrier. For this reason, we have conducted the DSC analysis on sputtered samples. To adjust the numerical model, the results obtained from the simulations were compared with those of the experimental measurements from the high-speed camera detecting of the reaction front velocity and the temperature measurements by a high-speed pyrometer.

## 2. Materials and Methods

**Figure 1:**
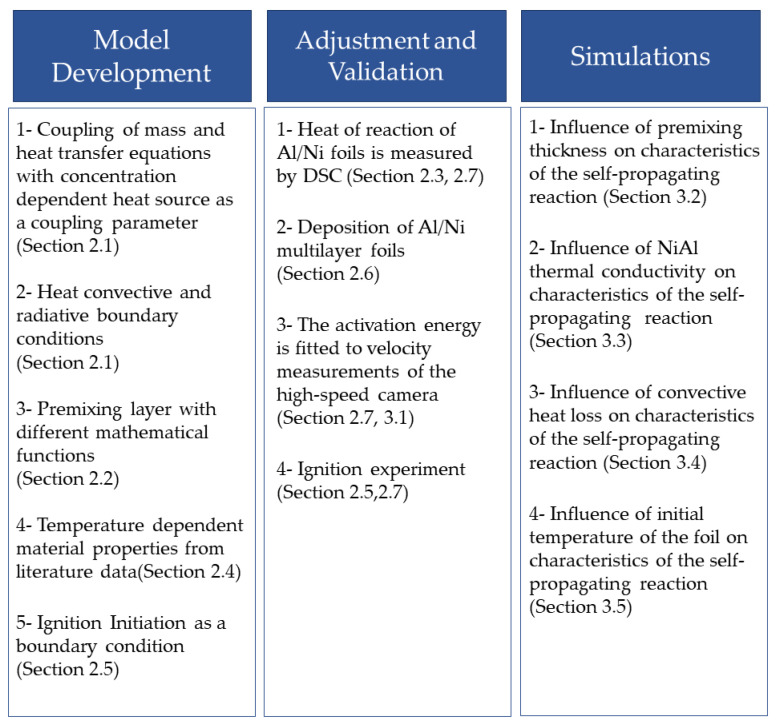
Summary of the current study.

The summary of the conducted research is shown in Figure 1. In the first step, the alternating Al and Ni layers were deposited by magnetron sputtering to total multilayer thickness of 1 µm. After that, free-standing multilayers were fabricated using the mechanical exfoliation process. The formed free-standing foils were ignited using an electrical ignition spark between two measurement tips, placed on the surface of the reactive multilayer foils. The propagation velocity of the reaction front and the time-temperature behavior were recorded using a high-speed camera and a high-speed pyrometer, respectively. Free-standing thin films were additionally measured via DSC to obtain the total reaction enthalpy (heat release) during the reaction with slow heating rates. The obtained data were compared to the results of the simulations of the propagation velocity and the time–temperature dependence to adjust and validate the simulation model.

### 2.1. Model Description

**Figure 2:**
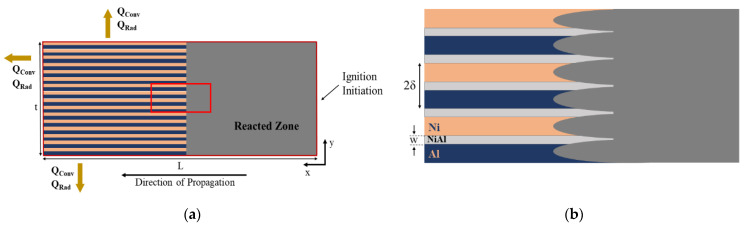
(**a**) Schematic of the implemented model. *Q_conv_* and *Q_rad_* are convective heat loss and radiative heat loss (**b**) enlarged view of the selected red area in (**a**), where w is the intermixing thickness of the multilayers and 2δ is the bilayer thickness.

Schematic of deposited Ni and Al layers are illustrated in Figure 2a. The length (*L*) of the model should be long enough to ensure that the finite size of the numerical domain is not affecting the characteristics of the self-propagating reaction [35]. On the other hand, it should be short enough to reduce computational costs. Therefore, the length of the multilayers is taken as 200 µm, to maintain a homogenous reaction front at different time steps. In Figure 2b, the formation of the reaction front and the intermixing zone of NiAl, which will be discussed later, is represented.

A two-dimensional numerical model is implemented characterizing the self-propagating diffusion reaction of Ni and Al multilayers. The computational domain and also the thermal boundary conditions, such as convective and radiative heat losses are represented in Figure 2a. Mass transfer equation is coupled with heat transfer equation with a heat source term represented in the following:

(1) $$\frac{\partial C}{\partial t}=\nabla \;\left(D\left(T\right)\;\nabla C\right),\;$$

Equation (1) is the mass transfer equation where C is atomic species concentration of Ni and Al, and D is the diffusion coefficient which is described in Equation (2), where $D_{0}$ is the pre-exponential coefficient and $E_{a}$ is the activation energy, *R* is the universal gas constant, and *T* is the temperature. To perform the calculations, $D_{0}$, is taken from [29].

(2) $$D=D_{0}\exp\left(-\frac{E_{a}}{R\;T}\right),\;$$

Equation (3) is the heat transfer equation, where ρ is the temperature dependent density, *C_p_* is the temperature dependent specific heat capacity, *K* is the temperature dependent thermal conductivity, and *Q*(*C*) which is taken from [35] is the concentration dependent heat source, which couples the mass and heat transfer equation mentioned earlier and it is defined in Equation (4), where $H_{rxn}$ is the heat of reaction.

(3) $$\rho \left(T\right)\;C_{p}\left(T\right)\;\frac{\partial T}{\partial t}=\nabla \;\left(K\left(T\right)\;\nabla T\right)+Q\left(C\right),\;$$

(4) $$Q\left(C\right)=-\rho \left(T\right)H_{rxn}\frac{\partial C^{2}}{\partial t},\;$$

The convective heat loss can theoretically be calculated from Equation (5) where *h*, is the convection coefficient, $T_{\infty }$, is the ambient temperature, and *A* is the surface area from where the heat is transferred.

(5) $$Q_{conv}=hA\left(T_{\infty }-T\right),\;$$

### 2.2. Premixing and Concentration Profiles

**Figure 3:**
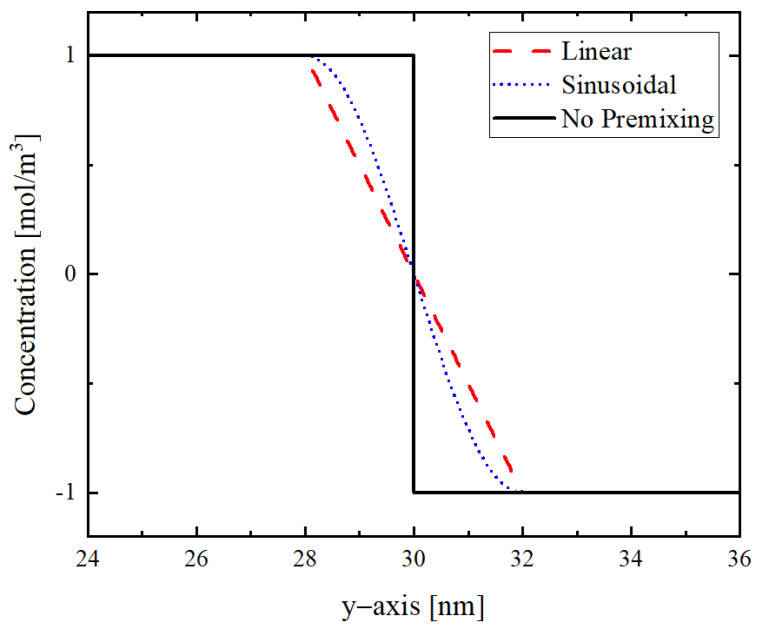
Possible mathematical concentration profile models of the intermixed zone at the bilayer interface and between the bilayers.

Al and Ni atoms are already intermixed during the sputtering process [20]. In Figure 3, the imposed concentration profile is plotted, as described; the primary concentration of Al and Ni and intermixed NiAl is considered 1, −1, and 0, respectively. The intermixed zone is implemented as linear and sinusoidal concentration profiles, as proposed in [35]. In an ideal case (no pre-intermixing), the concentration profile forms a step function as represented in Figure 3. The concentration distributions with a finite transition layer account for the existence of a premixing layer.

In [36], the prediction of the premixing thickness of 4 nm is reported, which is also comparable with the DSC analysis done in this work. This will be elaborated in detail in the next subsections.

### 2.3. Heat of Reaction

Experimental and theoretical studies have been performed to measure and calculate the enthalpy of formation of the B2 NiAl phase [30,37,38]. As reported by [30], the enthalpy of formation of NiAl varies in the range of −57.2 to −80 kJ g-atom^−1^. The negative sign is showing that the reaction is exothermic.

Due to the premixing of Ni and Al phases before the initiation of the self-propagating reaction, the heat, which is generated from the multilayer system decreases with the increasing thickness of the premixing layer *w* as is illustrated in Figure 2b. This effect is well investigated in [9]. According to [9], the dependence of the heat of reaction, on the bilayer thickness and the premixing thickness can be described by Equation (6).

(6) $$H_{rxn}=H_{f}\;\left(\;1-\frac{w}{\delta }\;\right),\;$$

where $H_{rxn}$ is the heat of reaction and $H_{f}$ is the enthalpy of formation of NiAl from its constituent phases.

**Figure 4:**
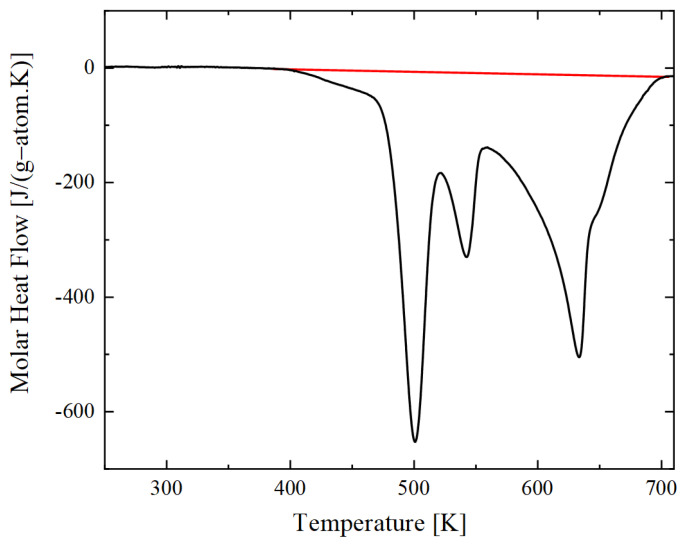
Molar heat flow curve of free-standing thin films for an equiatomic Al/Ni multilayer with a 50 nm bilayer periodicity measured with a constant heating rate of 0.333 K s^−1^. The red line displays the upper limit for the area of integration that was used for the determination of the total enthalpy release.

The DSC measurement (see Section 2.7) conducted in this study is shown in Figure 4. The measured enthalpy of reaction of deposited Al/Ni multilayers is −55.01 kJ g-atom^−1^ (where 1 g-atom = 1 mole of atom) or −1284 J g^−1^. This value, however, is measured by DSC for a constant slow heating rate of 20 K min^−1^ (0.333 K s^−1^), and the reaction starts at around 400 K and is completed at 700 K; it means that the reaction during the DSC annealing occurs exclusively in the solid-state. When the reaction is ignited at higher temperatures, where Al is in the molten state, the enthalpy of the reaction is expected to be more negative by at least 7 kJ mol^−1^ [30,39]. With a prediction of a premixing thickness of 4 nm by [36] and Equation (6), we can estimate the enthalpy of formation of the NiAl phase being approximately −65.5 kJ g-atom^−1^ or −1528 J g^−1^, which is in good agreement with literature data reported in [30]. This value is taken to perform the numerical study.

### 2.4. Material Properties

**Figure 5:**
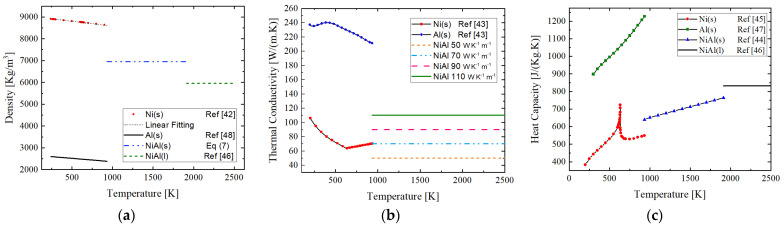
(**a**) Temperature dependent density of Al and Ni in solid-state; dotted line shows the linear fitting of data extracted from [42]. For temperatures greater than 933 K, the density of NiAl in the solid and liquid state is plotted. (**b**) Temperature dependent thermal conductivity of Nickel and Aluminum in solid-state taken from [43]. It is combined with different values of thermal conductivity of product (NiAl). (**c**) Temperature dependent heat capacity of Ni, Al in solid-state, and NiAl in both solid and liquid state extracted from [44,45,46,47].

The thermal properties, affecting the diffusion-driven reaction are thermal conductivity, density, and specific heat capacity of individual compounds and final product as well as intermetallic phases which are formed during the self-propagating reaction. The precision of implementation of these properties within the numerical model, makes simulation to be close to the real phenomenon of the Ni and Al reaction. For this, temperature dependent thermal properties are implemented. In [40], it was shown that NiAl crystalline phase is formed in 300 ns, after passing of the reaction front. It is known that the diffusion rate after melting of Al at 933 K significantly increases due to liquid–solid diffusion instead of solid–solid diffusion. Therefore, the reactant mixing rate dramatically rises [41]. In the current study, it is assumed that the dominating phase formed at the propagation front is NiAl; thus, we have adapted material properties and combined them as described in Figure 5. The isotropic material condition is assumed in this study, meaning that material properties are independent of direction.

The density of NiAl in the solid phase is estimated using Equation (7) as represented in Figure 5a, where *M* is the molar mass and ρ is the density of the compound.

(7) $$\rho_{NiAl}=\frac{M_{Ni}\;\rho_{Ni}+M_{Al}\rho_{Al}}{M_{NiAl}},\;$$

The Ni density data were extracted from [42] and linearly fitted. The fitting equation is described in Equation (8).

(8) $$\rho_{Ni\;}=9028.1-0.44925T\;T<T_{m,Ni},\;$$

where *T* is the temperature in Kelvin, ρ is the density in kg m^−3^, and *T_m,Ni_* is the melting point of Ni which is 1728 K. The Aluminum density was extracted from [48] and described in Equation (9).

(9) $$\rho_{Al}=2378-0.3111\left(T-933\right)\;T<T_{m,Al},\;$$

The melting point of Al is *T_m,Al_* = 933 K. Due to large deviations in thermal conductivity data at high temperature from the literature within the range of approximately 50–110 W m^−1^ K^−1^, we have studied the effect of different thermal conductivity values within this range on the velocity of reaction front and reaction temperature [43,46,49]. This is indicated in Figure 5b.

### 2.5. Ignition Initiator

Ignition is obtained by imposing initial energy, which starts the self-propagating reactions in the deposited multilayers. The possibilities for ignition are laser pulses, mechanical impact, electrical spark, or uniform heating of the foils [50]. The last three methods of ignition were studied in [33], where the threshold of ignition energy in form of energy density was reported for different ignition techniques. Igniting with laser pulses is studied in [51] for the Al/Pt system. In the current work, free-standing multilayer foils were ignited by the electrical current at 16 V. In the numerical model, heat pulse of $10^{11}$ W m^−2^ for 1 µs is applied on one side of the foil, to ensure the initiation of self-propagating ignition, while having insignificant thermal effects induced by the igniter.

### 2.6. Al/Ni Multilayer Deposition

The Ni and Al multilayers were prepared by magnetron sputtering using high purity Al and Ni targets in the PVD-Cluster 400 ES. Alternating layers of Ni and Al, with layer thicknesses of 20 nm and 30 nm for Ni and Al, respectively, were repeatedly deposited at room temperature onto p-type Si(100) substrates, covered with a native silicon dioxide layer, until the overall film thickness of 1 µm was reached. The starting layer was Al. The Ni and Al thicknesses were chosen to achieve a nearly 1:1 atomic ratio of the two elements in the bilayer. In all of these layers, the bilayer thickness was 50 nm.

### 2.7. Ignition and Measurements

**Figure 6:**
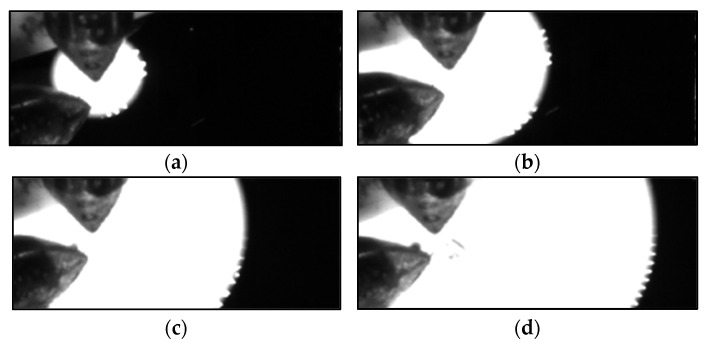
High-speed camera measurement. (**a**) At the time of ignition initiation, (**b**) t = 0.1 µs, (**c**) t = 0.2 µs, and (**d**) t = 0.3 µs.

The free-standing multilayer Al/Ni foils were ignited electrically by placing two measurement tips on the surface of the foils at the edge of the samples. The distance between the tips was 1 mm. The ignition was initiated by applying a voltage of 16 V between the tips. The velocity of the reaction front was recorded using a high-speed camera FASTCAM SA-X2 with a frame rate of 50,000 fps. The reaction temperature was measured using a high-speed KLEIBER-Pyrometer Pyroskop 840. The focus of the pyrometer was set to a predefined location and the temperature-time dependence was measured when the reaction front was passing it (see Figure 6).

In separate experiments, the heat of reaction of the Al/Ni multilayer foils was determined by DSC measurements using a power-compensated Perkin Elmer Differential Scanning Calorimeter 8500 using Al crucibles under a constant flow of high-purity Ar (99.99 mol. %) of 20 mL min^−1^ (see Section 2.3). The samples were heated with a constant rate of 0.333 K s^−1^ from 200 to 823 K. A second run with the reacted material under identical conditions allowed for determining the baselines, which were subtracted from the first up-scan. The calorimeter was calibrated by measuring the melting temperatures and melting enthalpies of In and Zn [52]. Multiple pieces of the thin films have been stacked to reach a sufficient total mass for a good signal to noise ratio.

**Figure 7:**
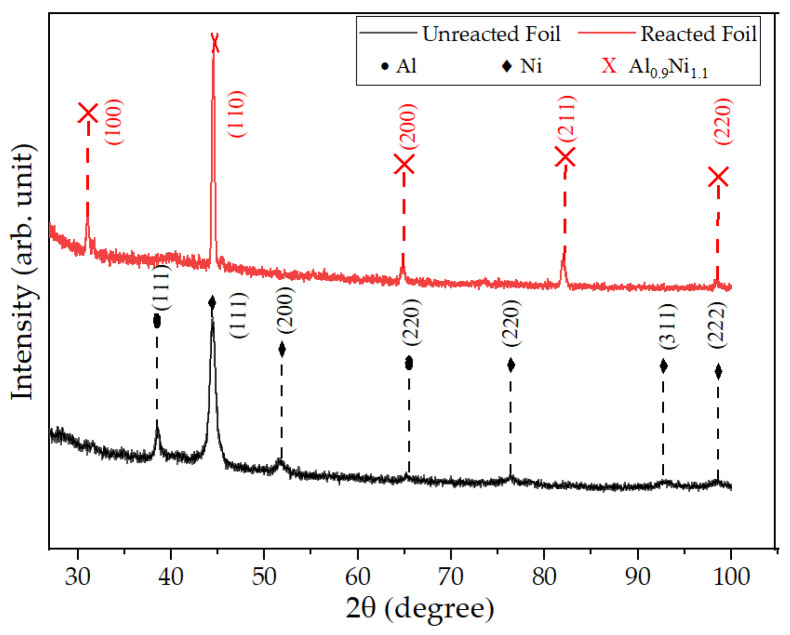
X-ray Diffraction analysis of free-standing Al/Ni multilayer foils before and after the self-propagating reaction.

The phase identification for the reacted and unreacted Al/Ni free-standing foils was carried out by X-ray diffraction (XRD) (Bruker D5000 Theta-Theta X-ray diffractometer) with Cu-Kα (1.5406 Ȧ) radiation at 40 kV and 40 mA in Bragg–Brentano working mode. Figure 7 depicts the XRD patterns of unreacted and reacted free-standing foils. It shows that before the reaction, the main peaks are belonging to Al (PDF 01-1179) and Ni (PDF 01-1179) and indicate the dominating presence of an fcc (111) orientation of Al and Ni elements in the unreacted free-standing foils. After the reaction, significant changes in the structure are shown by the elemental Al and Ni peaks disappearing and instead, exhibiting the B2 Al_0.9_Ni_1.1_ (PDF 44-1185) phase [53].

No additional phases were found which gives proof of the formation of the Al_0.9_Ni_1.1_ phase after the reaction. This Ni rich NiAl phase has already been observed due to changes in the composition of the grains in already existing phases [54].

### 2.8. Foil Formation

For the fabrication of the free-standing foils, a technique was used which is widely applied to exfoliate and transfer two dimensional materials to foreign substrates. It is the so-called dry transfer technique [55]. For the formation of the free-standing foils, the first step of this technique was used in a modified form. Specifically, a Kapton tape was mounted to the edge of the Si(100) substrate covered with the Al/Ni multilayer. Subsequently, the Al/Ni multilayer was exfoliated from the substrate. The Kapton foil was not removed from the foil edge, but used to mount the foil to the ignition set-up.

## 3. Results and Discussion

### 3.1. Adjustment of Model Parameters

The parametric validation of the developed model was conducted by relating the dependence of the simulated reaction propagation front velocity for a given width of the intermixing zone between the constituent layers of the bilayer stacks to the experimentally determined reaction front velocity. The correlation of the simulated and the experimentally determined reaction front velocity allows the determination of the *E_a_*. In this approach, it was assumed that the width of the intermixing zone is equal for all interfaces.

**Figure 8:**
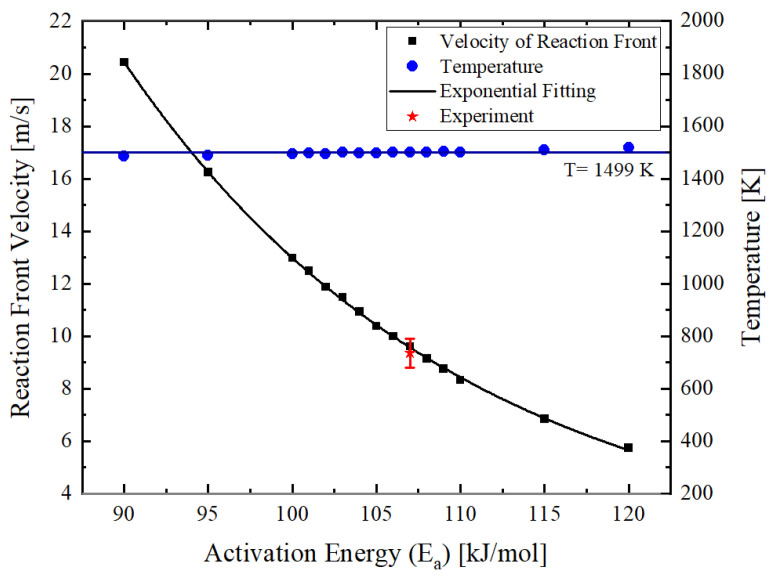
Effect of the activation energy of diffusion on the reaction front velocity and the reaction temperature at the reaction front. The width of the intermixing zone was set to 4 nm.

Figure 8 illustrates the effect of the *E_a_* on the reaction front velocity and the reaction front temperature. The reaction front temperature is independent of the activation energy and is equal to 1499 K. The measured temperature by high-speed pyrometer showed the maximum temperature of 1396 K. The deviation of temperature results of simulation and measurement is about 7%. This difference can be due to the finite integration time of the pyrometer used for the temperature measurements. Furthermore, it has to be mentioned that in the model, the interface roughness and waviness were not taken into account. The morphological real structure of the interface can influence the reaction temperature, as it acts as an effective medium and effectively increases the intermixing zone.

The reaction front velocity decreases with increasing the activation energy *E_a_* following an exponential rule introduced in [56] and later implemented by [9,36] as well. The application of the correlation procedure yields an $E_{a}\;$= 107 kJ mol^−1^. The obtained value agrees with the results obtained in [57,58]. For the following numerical investigations, this value was used for the activation energy.

### 3.2. Influence of Premixing

**Figure 9:**
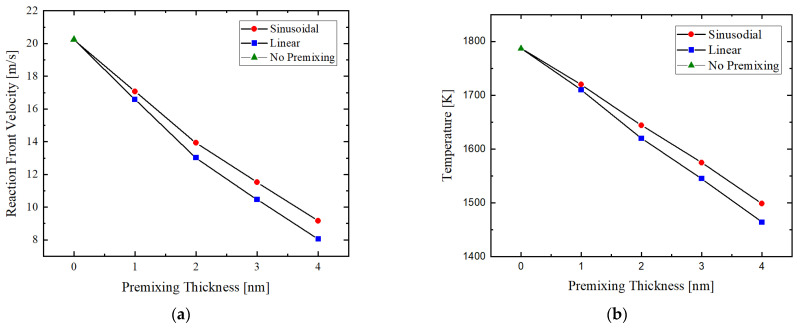
The effect of sinusoidal and linear premixing zone (explained in Section 2.2) on (**a**) the reaction front velocity and (**b**) the reaction temperature at the reaction front, where $E_{a}=107\;{\mathrm{kJ}\;\mathrm{mol}}^{-1}$, $D_{0}=2.18\times 10^{-5}\;m^{2}s^{-1}$ and heat of reaction is calculated by Equation (6). (Lines are drawn to guide the eyes).

The influence of the thickness of the premixing zone at the interfaces between the two constituent materials is shown in Figure 9. Linear concentration profiles have a larger impact on both characteristics of the self-propagating reaction. Increasing premixing thickness causes a lower diffusion rate at the interface and a decrease in heat of reaction of Ni and Al which results in a lower propagation velocity of the reaction front. This is also mentioned in [35] and it is in good agreement with the effect of premixing (sinusoidal and linear) on the velocity of the reaction front and reaction temperature conducted in this study.

### 3.3. Influence of NiAl Thermal Conductivity

**Figure 10:**
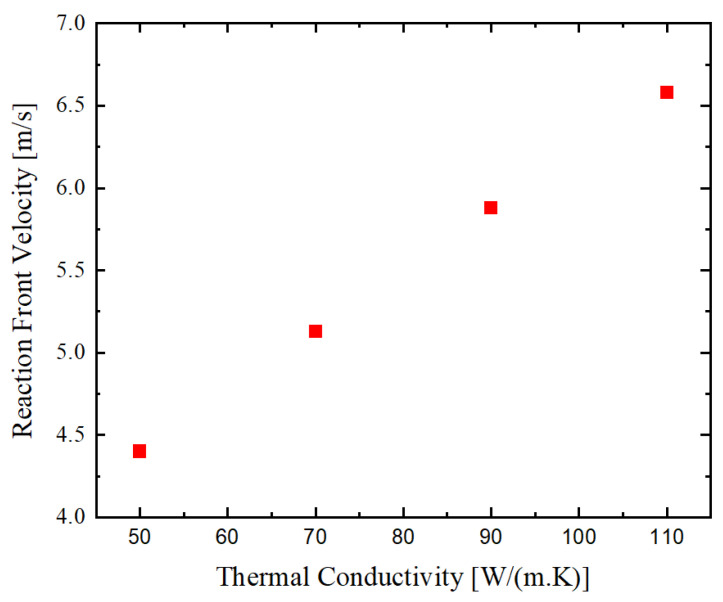
Influence of thermal conductivity of the final reaction product on the self-propagating reaction front velocity.

As mentioned in Section 2, the thermal conductivity of the reacting materials and the reaction product impacts the characteristics of the self-propagating reactions. On the other hand, the thermal properties of the initial materials in the bilayer stack and reaction product are affected by their real structures and therefore, depend on defect densities and composition. For the estimation of the impact of the thermal conductivity on the reaction front velocity, this parameter was varied. The results of the carried-out simulations are shown in Figure 10. According to the obtained results, the reaction front velocity increases nearly linear with the increase of the thermal conductivity of the final reaction product NiAl.

### 3.4. Influence of Convective Heat Loss

**Figure 11:**
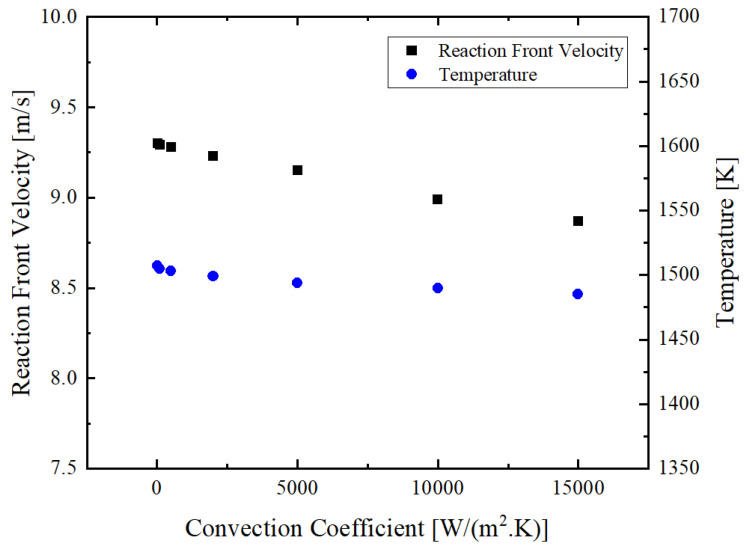
Effect of changing the ambient condition (convective coefficient) on the reaction front velocity and the temperature at the reaction front.

Changing the ambient condition can cause change in the convective coefficient and consequently, affects heat loss from the multilayer foil to the environment (see Equation (5)). In Figure 11, the dependence of reaction front velocity and temperature at the reaction front on the convection coefficient is illustrated. Calculations showed in Figure 11 that for increasing convection coefficients from 5 to 15,000 W m^−2^ K^−1^, the propagation reaction front velocity decreases slightly. The relative decrease in the values of the reaction front velocity and the reaction temperature was obtained to be about 5 and 1.5%, respectively.

Therefore, increasing heat transfer coefficients causes a reduction in both characteristic values of the self-sustained reaction. Table 1 represents the convection coefficients related to these environmental conditions. From Table 1 and the calculations for different convection coefficients, we can conclude that multilayer foils behave similarly in an aqueous environment as they do in air.

### 3.5. Influence of Initial Temperature

**Figure 12:**
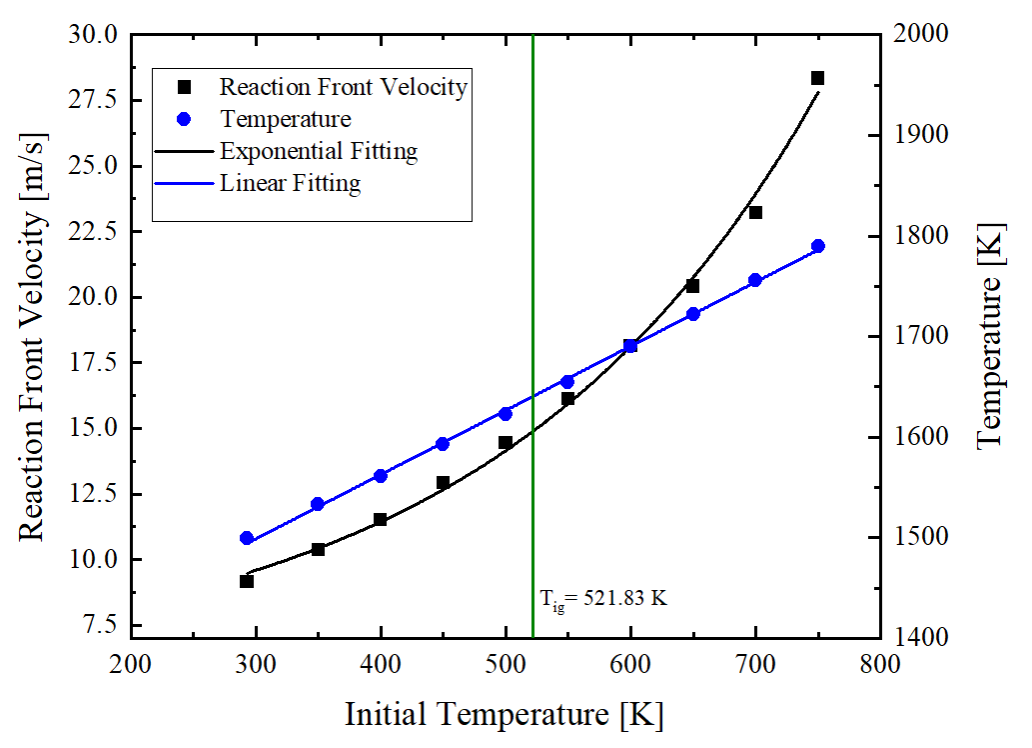
Influence of initial temperature of the reactive multilayer foil on the propagation reaction front velocity and temperature.

In the previous subsections, the influence of the thermal conductivity and the heat loss conditions on the reaction front velocity and the reaction front temperature were studied. In Section 2.4, it was shown that the properties of the materials are affected by the temperature. Therefore, it is of interest to study the effect of the initial temperature of the multilayer foils. In [50], an ignition temperature of 521 K was measured on a hot plate for the solid-state reaction of Al/Ni multilayer foils. The results of the calculations are summarized in Figure 12. It is predicted that, with increasing the initial temperature of the foils from room temperature up to the ignition initiation temperature, we are able to increase the reaction front velocity within the range of 5 m s^−1^ and the temperature at the reaction front within the range of 100 K (see Figure 12).

The increase of the reaction front velocity follows an exponential function whereas the reaction temperature obeys a linear law. Therefore, the initial temperature allows tuning both of these characteristic properties of the self-propagating reactions in reactive multilayer foils.

## 4. Conclusions

A two-dimensional numerical model was developed to investigate and analyze the influence of environmental variables and material properties on reaction front velocity and reaction temperature of reactive Ni and Al multilayer foils. For this, mass and heat transfer equations were coupled with a concentration dependent heat source as a coupling parameter. The interdiffusion of Ni and Al reactants and the formation of the final NiAl intermetallic alloy were considered. The boundary conditions of the interaction of the reactive multilayer system were determined by the heat dissipation conditions of a viscous medium, i.e., radiation, heat conduction, and convective losses have been chosen. Basic assumptions of the current study were as follows: (1) stoichiometric reaction of Al and Ni, (2) only the NiAl phase exists at temperatures higher than melting temperature of aluminum, and (3) isotropic temperature dependent material properties. It is shown that with increasing thermal conductivity of the final product of the self-propagating reaction, the reaction front velocity increases. Convective cooling has an insignificant effect on reaction characteristics. It is predicted that Ni and Al multilayer foils can react in liquid as well as in an air environment. The characteristics of the reaction can be controlled, imposing the multilayer foils at different initial temperatures. With the increase of the initial temperature of the reactive multilayer systems, the velocity and the reaction temperature will increase.

## Figures and Tables

**Table 1:** Convection Coefficient Examples (taken from [59]).

Convection Type	Convection Coefficient h (W m^−2^ K^−1^)
Air, Free Convection	2.5–25
Air, Forced Convection	10–500
Liquid, Forced Convection	100–15,000

## Data Availability

The data that support the findings of this study are available from the corresponding authors, M.B. and J.P., upon reasonable request.
